# Data-Efficient Sensor Upgrade Path Using Knowledge Distillation

**DOI:** 10.3390/s21196523

**Published:** 2021-09-29

**Authors:** Pieter Van Molle, Cedric De Boom, Tim Verbelen, Bert Vankeirsbilck, Jonas De Vylder, Bart Diricx, Pieter Simoens, Bart Dhoedt

**Affiliations:** 1 IDLab, Department of Information and Technology, Ghent University, 9052 Gent, Belgium; cedric.deboom@ugent.be (C.D.B.); tim.verbelen@ugent.be (T.V.); bert.vankeirsbilck@ugent.be (B.V.); pieter.simoens@ugent.be (P.S.); bart.dhoedt@ugent.be (B.D.); 2 Barco Healthcare, Barco N.V., 8500 Kortrijk, Belgium; jonas.devylder@barco.com (J.D.V.); bart.diricx@barco.com (B.D.)

**Keywords:** deep learning, knowledge distillation, cross-modal distillation, sensor upgrade, skin lesion classification, multispectral imaging

## Abstract

Deep neural networks have achieved state-of-the-art performance in image classification. Due to this success, deep learning is now also being applied to other data modalities such as multispectral images, lidar and radar data. However, successfully training a deep neural network requires a large reddataset. Therefore, transitioning to a new sensor modality (e.g., from regular camera images to multispectral camera images) might result in a drop in performance, due to the limited availability of data in the new modality. This might hinder the adoption rate and time to market for new sensor technologies. In this paper, we present an approach to leverage the knowledge of a teacher network, that was trained using the original data modality, to improve the performance of a student network on a new data modality: a technique known in literature as knowledge distillation. By applying knowledge distillation to the problem of sensor transition, we can greatly speed up this process. We validate this approach using a multimodal version of the MNIST dataset. Especially when little data is available in the new modality (i.e., 10 images), training with additional teacher supervision results in increased performance, with the student network scoring a test set accuracy of 0.77, compared to an accuracy of 0.37 for the baseline. We also explore two extensions to the default method of knowledge distillation, which we evaluate on a multimodal version of the CIFAR-10 dataset: an annealing scheme for the hyperparameter α and selective knowledge distillation. Of these two, the first yields the best results. Choosing the optimal annealing scheme results in an increase in test set accuracy of 6%. Finally, we apply our method to the real-world use case of skin lesion classification.

## 1. Introduction

Thanks to the rise of powerful parallel processing units, deep neural networks, such as large convolutional neural networks, have become state-of-the-art in image classification [1,2,3,4,5]. Simultaneously, a lot of research is being put into improving existing sensor technologies and developing new ones, such as novel multispectral cameras or millimeter wave radar sensing technologies. This results in more advanced and more capable data modalities. An other example of this are depth cameras, which do not only capture visual information (RGB data), but also capture depth information. Since deep neural networks achieve high performance on regular camera images, they are increasingly applied to these new data modalities as well. However, an important factor in the success of neural networks is the availability of large scale datasets, such as ImageNet [6]. When deploying a new or updated sensor technology, equivalently large datasets are not (yet) available, nor do the original data modalities necessarily map to the new ones. Therefore, we are left with a choice: either stick with the original data modality until a large enough dataset becomes available that uses the new technology, or transition to the new data modality, thereby potentially hurting classification performance because of poor data availability. This delays the time to market and adoption rate of novel sensor technologies, although these might improve performance in the long run. Knowledge distillation [7] can alleviate this hard transition, by leveraging the availability and performance of the original data modality to train a neural network on the new data modality.

Knowledge distillation is the process of compressing the knowledge of a large, cumbersome neural network (the “teacher”), or an ensemble of networks, into a smaller neural network (the “student”). The teacher network is trained on a sufficiently large train set, optimized to generalize well on unseen data. The student network is then trained on either a different train set, which could very well exist of unlabelled data, or using the same train set that was used to train the teacher network. During training of the student network, the class probabilities generated by forwarding the train set through the teacher network are used as soft targets for the student network. In case of an ensemble of teacher neural networks the individual probabilities for a given class can be aggregated to provide a soft target for this particular class. If the train set is labelled, the ground truth labels can be used during training as well. In this case, the soft targets provide additional information during training.

Knowledge distillation can be generalized across modalities, as would be required when upgrading sensor technologies. In cross-modal distillation [8], the knowledge from a teacher network that was trained on a large, annotated dataset in one modality, is distilled into a student network by training on another, potentially unlabelled, dataset in a different modality. More specifically, this method relies on the availability of paired samples, containing both modalities. When upgrading sensors, however, only a limited body of paired samples is available. In this work, we evaluate cross-modal distillation for this setting specifically, when little data is available in the new data modality. In particular, we investigate knowledge distillation as an alternative to a hard transition from the original data modality to the new modality, and look at the evolution in performance as more data of the new modality becomes available. Additionally, we research the impact of class imbalance on the system, which is often overlooked in other work. Finally, we apply our method to the real-world use case of skin lesion classification.

The remainder of this paper is structured as follows. In Section 2 we give an overview of related work. In Section 3 we present (cross-modal) knowledge distillation, and how we apply it to our use case of upgrading sensors. We describe the datasets we use to evaluate our approach in Section 4, followed by the experimental results. We discuss these results in Section 5. We end this paper with concluding remarks in Section 6.

## 2. Related Work

Following Hinton et al. [7], who distilled the knowledge of a large neural network to improve performance of a smaller neural network on the same test set (i.e., both the teacher and the student network were trained on the same data modality), Gupta et al. [8] applied knowledge distillation across data modalities, where the teacher network is trained on one modality, and the student is trained on a different modality. The authors trained a teacher network for the task of scene recognition on RGB images, and used this network to train a student network on a large body of unlabelled depth images. Since then, cross-modal distillation has been applied to various applications.

Audiovisual footage is ideal for this approach, having synchronized audio and video available. Albanie et al. [9] distilled the knowledge from a teacher, pre-trained on a large body of audiovisual samples, for the task of emotion recognition in audio-only data, thereby eliminating any data redundancy contained in video. On the other hand, Aytar et al. [10] used a teacher network trained for visual scene recognition, to improve training of a student network for the task of acoustic scene classification. Zisserman et al. [11] and Zhao et al. [12] used speech samples as additional supervision for the task of visual speech recognition. Ren et al. [13] further improved on this concept, by introducing curriculum learning.

Thoker and Gall [14] transferred the knowledge of RGB videos to 3D skeleton poses, for the task of action recognition. Crasto et al. [15] and Dai et al. [16] applied cross-modal distillation to the same task, by augmenting a student network, trained on RGB data, with knowledge from a teacher network, trained on optical flow data.

The drawback of these approaches is that they require a large body of paired samples, which usually is not available when upgrading sensors. In contrast to the previously mentioned works, we evaluate cross-modal distillation when little data is available in the new modality, as would be the case when upgrading to a new sensor.

## 3. Materials and Methods

### 3.1. Knowledge Distillation

In classification tasks, a neural network outputs a discrete probability distribution p in which $p_{i}$ is the output probability for class *i*. This is achieved by applying the softmax function to the output logits z:

(1) $$p_{i}\left(z,T\right)=\frac{\exp(z_{i}/T)}{\sum_{j}\exp\left(z_{j}/T\right)}$$

in which *T* is a temperature parameter, usually set to 1. When a higher temperature value is used, the absolute differences between the output probabilities of different classes become less pronounced, thereby producing a distribution that is less peaked.

Knowledge distillation [7] uses the probability distribution generated by a large teacher neural network—or an ensemble of networks—as soft targets to train a small student neural network. Usually a high temperature is used to soften the probability distribution. The resulting *distillation loss* $L_{D}$ is then given by the cross-entropy between the probability distributions generated by the student and teacher networks:

(2) $$L_{D}=\sum_{i}-p_{i}\left(z_{t},T\right)\log\left(p_{i}\left(z_{s},T\right)\right),$$

where $z_{t}$ and $z_{s}$ are the logits of the teacher and the student respectively.

When ground truth labels are available, these can be used in conjunction with the soft targets provided by the teacher network. In this case, the *student loss* $L_{S}$ is also a cross-entropy loss defined as follows:

(3) $$L_{S}=\sum_{i}-y_{i}\log\left(p_{i}\left(z_{s},T\right)\right),$$

where y is the one-hot vector encoding the ground truth label. The total loss is then given by the convex combination of both the distillation loss and the student loss:

(4) $$L=\alpha L_{D}+\left(1-\alpha \right)L_{S},$$

where α is a hyperparameter, in addition to the temperature parameter *T*.

### 3.2. Sensor Upgrade Path

In this paper, we apply knowledge distillation to the problem of switching data modalities, for example, when upgrading sensors. We consider the scenario where a sensor is to be replaced by a more recent (or powerful) one. We also assume that we have a large dataset at our disposal that was captured using the original sensor. Using this dataset, a neural network was trained for a given classification task. We now want to use the upgraded sensor to solve the same task with equal or better classification performance compared to the original sensor. This poses two challenges. First, enough new data needs to be captured in the new data modality to train a neural network that performs similarly compared to the original network. This could take a long time, especially when certain events occur with a low frequency. Second, the newly captured data needs to be labeled manually, which is a labour-intensive task. As we will show in the following, by using knowledge distillation we reduce the need for a large dataset in the new data modality, thereby alleviating both problems. Since a small body of paired samples is still required, ideal systems for our approach are systems that by design capture data in multiple modalities. Examples of such systems are camera modules with multiple sensors, or multispectral sensors that also capture an RGB image. In other cases, where the new sensor is isolated, our approach can still be applied, by co-deploying the original and the new sensor, jointly capturing the paired training samples.

**Figure 1:**
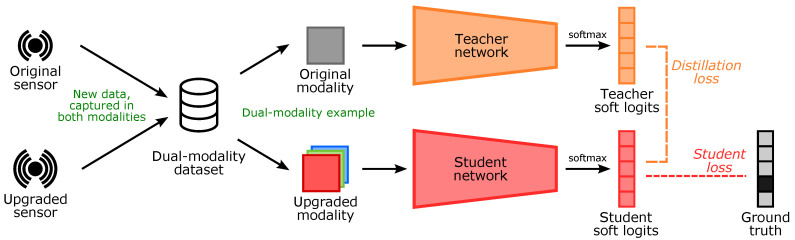
Upgrading to a new sensor. During transition, a small dataset is captured using both sensors. Using this dataset, a student network is trained on the new data modality, with the help of the original network, now functioning as teacher.

Figure 1 gives an overview of our method. We use both sensors, and capture and annotate an initial dual-modality dataset, where the same event is captured in both modalities. We employ the original neural network as the teacher, to bootstrap learning for the new student network. To train the student network, we take a data sample (containing both modalities) and pass the original modality through the (previously trained) teacher network to obtain teacher soft logits. Next, we pass the new modality through the student network, obtaining student soft logits. Using both the teacher soft logits and the student soft logits, we calculate the distillation loss. We compare the soft logits generated by the student to the ground truth labels to calculate the student loss. The total loss is then given by (4).

### 3.3. Extending Knowledge Distillation

In its default mode, the total knowledge distillation loss is given by (4), i.e., the weighted sum of both the distillation loss and the student loss, with a fixed hyperparameter α. We propose two extensions to this default mode. In a first extension, we no longer keep α fixed, but rather anneal its value during training. In the second extension, we will only use the teacher soft logits when the teacher’s classification of the given data example is correct. We will now go over the details of both extensions.

#### 3.3.1. Annealing α

By default, the student tries to mimic the output distribution of the teacher through the distillation loss. When ground truth labels are available, additional information can be provided during training through the cross-entropy between the student soft logits and these ground truth labels, i.e., the student loss. The α hyperparameter balances the amount of knowledge that is distilled from the teacher and the information provided by the ground truth labels. The teacher network helps to bootstrap the student network. Given enough training steps, the student will achieve similar performance for the new data modality compared to the teacher, as we will show in Section 4. However, it is possible that, at this point, by forcing the student to mimic the teacher, the student cannot fully utilize the additional information present in the new modality. In this case, the teacher actually hinders the student’s performance. To overcome this problem, we propose an annealing scheme for α, where its value is linearly annealed during training. This way, the influence of the teacher diminishes as training progresses. For a linear annealing scheme, the total loss at iteration *i* (out of a total of *n* iterations) is given by

(5) $$\begin{array}{cc} L & =\alpha_{i}L_{D}+\left(1-\alpha_{i}\right)L_{S},\\ \mathrm{with}\;\alpha_{i} & =\alpha_{0}-i\cdot \alpha_{step},\\ \alpha_{step} & =\frac{\alpha_{n}-\alpha_{0}}{n-1}, \end{array}$$

where $\alpha_{0}$ and $\alpha_{n}$ are the initial and final value for α, respectively.

#### 3.3.2. Selective Knowledge Distillation

Although a teacher is imperfect, distilling its knowledge still helps to bootstrap a student during training. The additional information from the teacher’s soft logits helps the student to differentiate between classes. Even when the teacher is wrong, these soft logits can still contain useful information. For example, a teacher might not clearly label an image of a car as such, thinking it is more similar to a truck. However, it might know for sure that the image does not contain a bird. However, after the bootstrapping phase, forcing a student to follow the teacher, in all cases, including the incorrect ones, could again hinder the student’s performance. To prevent this from happening, we propose a method called selective knowledge distillation, where, after an initial bootstrapping phase, the distillation loss is only used for the data examples for which the teacher label is correct. For the other examples—that are wrongly classified by the teacher—we only use the student loss, i.e., we explicitly set α to zero.

## 4. Experiments and Results

### 4.1. Red Datasets

We first validate our approach using modified versions of two benchmark datasets. Next, we apply our method to a real-world use case, namely that of skin lesion classification.

#### 4.1.1. Benchmark Datasets

To validate our approach, we use modified versions of the MNIST [17] and CIFAR-10 [18] datasets. Modifications are applied as follows.

#### MNIST

**Figure 2:**
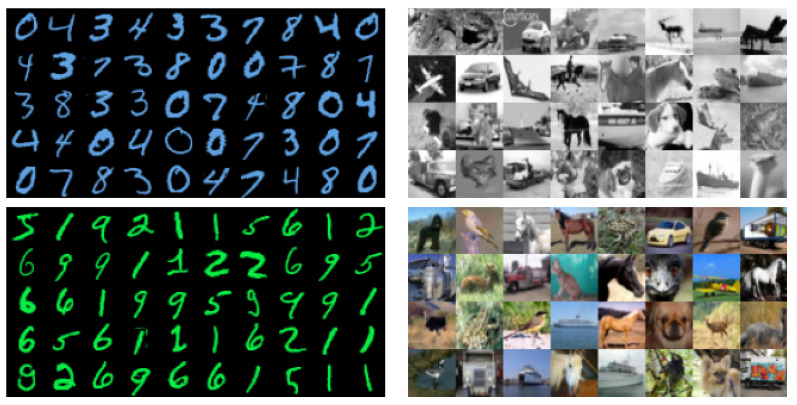
Examples of the images in the (modified) benchmark datasets. **Left:** The colored digits in the 2cMNIST dataset. These are generated by coloring the greyscale images in either blue or green (full details in text). When converted back to greyscale, all these examples have the same luminance. **Right:** Greyscale and color versions of the images in the CIFAR-10 dataset. Best viewed in color.

The MNIST dataset contains images of handwritten digits in black and white, i.e., greyscale images having only a single channel. We expand the number of channels to three by coloring the digits, thereby introducing one extra degree of freedom in the dataset, dubbed 2cMNIST. We use two colors: blue (hex value #61a4e4) and green (hex value #0aef46). All instances of a single class are colored using the same color. We have assigned colors to the digits by training a small neural network on the greyscale images, and evaluated the resulting confusion matrix. Digits that are difficult to distinguish on average, such as the “3” and “9”, get a different color. More specifically, we color the digits “0”, “3”, “4”, “7” and “8” blue, and the digits “1”, “2”, “5”, “6” and “9” green. We have chosen specific shades of blue and green, so that their greyscale versions have the same luminance, i.e., the colors are indistinguishable when converted to greyscale. Examples of the modified digits are given by Figure 2 (left).

#### CIFAR-10

The CIFAR-10 dataset consists of colored images, equally divided over ten classes. We cast this dataset as a dual-modality dataset by using both greyscale and color versions of these images. By decreasing the number of channels in an image from three to one, we reduce the amount of information contained in each image. Examples of greyscale and color images for the CIFAR-10 dataset are given by Figure 2 (right).

#### 4.1.2. Skin Lesion Classification

In 2020, skin cancer was the most commonly diagnosed cancer in the United States. Furthermore, of all cancers, melanoma skin cancer is the most deadly. However, when discovered early, survival rate can exceed 98% (depending on the stage) [19]. Since early detection is done visually, this is an ideal case for deep learning. In controlled settings, neural networks perform similarly to teams of trained dermatologists, in single image classification of skin lesions [20,21,22].

Currently, most research on skin lesion classification is done using white light imaging. This is, in part, due to the availability of a large public image repository, hosted by the International Skin Imaging Collaboration (ISIC). Additionally, this organization hosts an image classification challenge each year, introducing a curated train set for each challenge [23,24,25].

An alternative to white light imaging is multispectral imaging. Unlike white light imaging, where a skin lesion is only illuminated once using white light, in multispectral imaging, skin lesions are illuminated multiple times with a set of light sources having different wavelengths. This is beneficial, since light penetrates the skin differently for different wavelengths. This way, structures at different depths in the skin are revealed, possibly containing vital information for classification. Red light, for example, having a longer wavelength, penetrates deeper into the skin, highlighting different features than blue light, which has a shorter wavelength [26,27]. We apply our approach using a large dataset of white light images, and a smaller dataset of multispectral images. An overview of the data is given by the following paragraphs.

#### ISIC Challenge 2019 Data

We use data from the 2019 ISIC Challenge [24,28,29]. The dataset for this challenge consists of 25,331 images of common pigmented skin lesions, which we divide over 7 ground truth classes: melanoma, nevus, basal cell carcinoma, actinic keratosis/squamous cell carcinoma, benign keratosis, dermatofibroma, and vascular lesions. Images are scaled down to 400×400 pixels. We randomly split the images into a train set (24,064 images, roughly 95%) and a validation set (1267 images, roughly 5%).

#### Multispectral Images

We use a proprietary dataset of multispectral images of skin lesions, captured using a prototype of the Barco Demetra^®^ dermoscope after patients had given informed consent. For each lesion in the dataset, this device took both a white light image and a multispectral image. The multispectral images were constructed by illuminating skin lesions with light at different wavelengths, mostly within the visible spectrum (400 nm to 800 nm). For every spectral channel, *auto-exposure* was applied, maximizing the signal-to-noise ratio, while avoiding sensor saturation. In total, we use five spectral channels. The original images have a resolution of 3840×2160 pixels, and are scaled down to 400×400 pixels, after cutting out a square image (2160×2160 pixels) in which the lesion is positioned at the center.

In total, the Demetra dataset contains 6875 images. We divide these over the same seven ground truth classes as the ISIC dataset. The data is heavily imbalanced, as can be seen in Table 1. In our experiments, we randomly split the data in a train set (5156 images, roughly 75%), a validation set (687 images, roughly 10%), and a test set (1032 images, roughly 15%).

### 4.2. Bootstrapping Student Learning Using Knowledge Distillation

We start by validating our approach, using the different versions of the 2cMNIST dataset to simulate the transition between data modalities. Here, the greyscale images represent the original modality, of which an abundance of data is available. The colored images act as the new modality, containing additional information, useful for classification. First, we use a large dataset of greyscale images (the old modality) to train a teacher network. Next, we use a small dataset of colored images (the new modality) to train a student network, with knowledge distillation, and a baseline network, without knowledge distillation. By increasing the size of the dataset used to train the student and baseline networks, we simulate data additions in the new modality. Finally, we determine the experimental (approximate) upper bound of the classification performance by training an upper bound network using the same, large, dataset used to train the teacher network—albeit colored images this time.

All networks have the same architecture, consisting of two layers. The first layer is a convolutional layer, having four filters, and a kernel size of 3×3, followed by a ReLU non-linearity. The following and final layer is a fully connected layer that maps the hidden representation to the ten output classes.

To train the teacher and the upper bound network, we randomly sample 5000 images from the 2cMNIST train set, using the greyscale versions for the teacher, and the colored versions for the upper bound. For the student and baseline networks, we vary the size of the dataset, using 10, 50, 100, 250, 500, 750, or 1000 randomly sampled (colored) images. The student network is aided in training, by distilling knowledge from the now-fixed teacher network. The total loss for the student is given by (4). We use a value of 0.6 for hyperparameter α, and a temperature value T=8. All networks are trained for 2000 iterations, using a batch size of 64. Network parameters are updated using stochastic gradient descent, with a learning rate of 0.01. Evaluation is done using the appropriate version of all 10,000 images of 2cMNIST test set: the greyscale version for the teacher network, and the colored version for all other networks.

**Figure 3:**
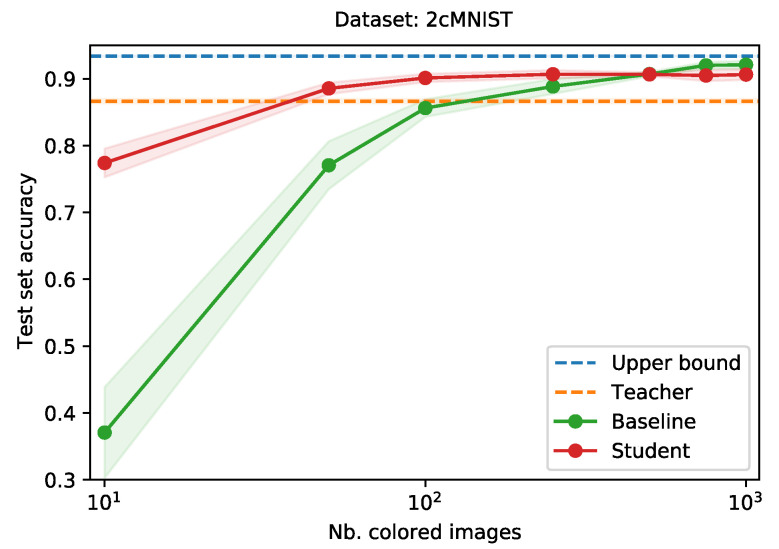
Comparison of the average test set accuracy when training a neural network, with and without knowledge distillation, as a function of the student train set size. Averages over three runs. Shaded areas indicate the standard deviation.

Figure 3 shows the performance of the teacher network (orange dotted line) and the student, trained with knowledge distillation (red) and baseline, trained without knowledge distillation (green), as a function of the their train set size. The upper bound is given by the blue dotted line. Especially when (extremely) little data is available in the new modality, applying our approach results in large gains in performance. Even when training the student with only 10 images, it realizes a test set accuracy of 0.77, when trained using knowledge distillation, as opposed to 0.37 for the baseline. This gap in performance decreases as the student train set grows.

The extra degree of freedom in the data, introduced by the colors, proves to contain a lot of information, as is shown by the difference in test set performance between the teacher network (0.86) and the upper bound (0.94). By training a student network using knowledge distillation, it leverages both the information provided by the teacher, as well as the extra information in the new data modality.

#### Imbalanced Data

The teacher, student and baseline networks are trained using a balanced train set (where the student and baseline only have access to a stratified subset of the dataset used to train the teacher). Real-world datasets, however, are often imbalanced. In the scenario where one or more events occur with a low frequency, collecting enough data in the new modality to train a neural network can take a long time. We simulate this scenario, with the assumption that sufficient data has been captured in the original modality to train a teacher network. We introduce a slight class imbalance in the train set used to train the teacher, and a much stronger imbalance in the train set for the student and baseline (resembling an early phase of gathering data in the new modality).

**Figure 4:**
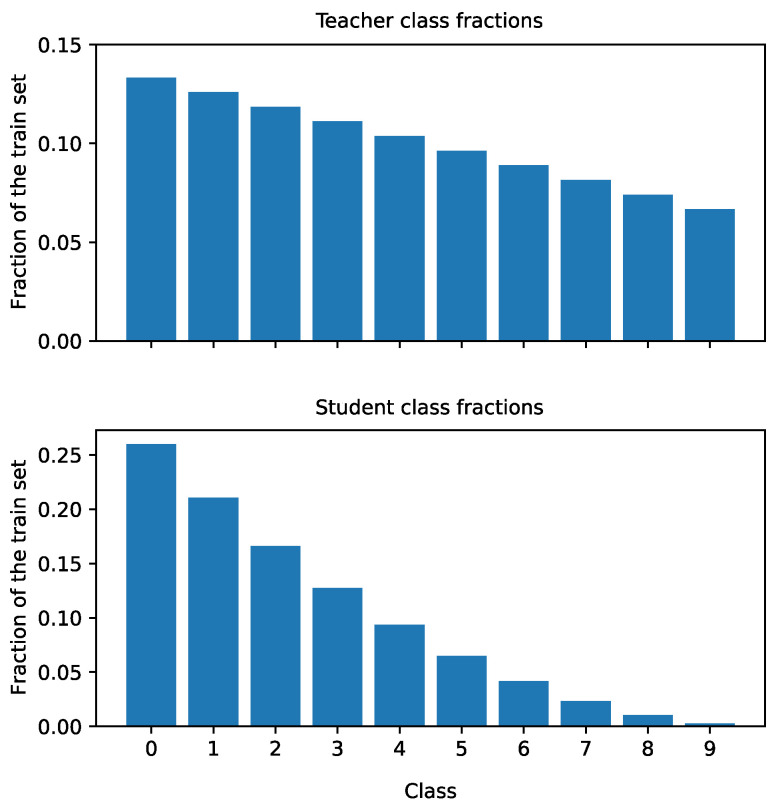
An overview of the available images per class, as a fraction of the total train set size. There is a slight class imbalance for the teacher (**top**), and a much heavier imbalance for the student and baseline (**bottom**).

To create an imbalanced train set for the teacher, we reduce the available images by taking only a fraction of the images for each class, using the fractions {1.00,0.94,⋯,0.56,0.50}. This way, after sampling, the train set will contain approximately twice as many images for the digit “0” as it will for the digit “9”. We use a similar strategy to create imbalanced train sets for the student and baseline, using the fractions $\{f={\left(1-i\right)}^{2}:$i∈{0,0.1,⋯,0.8,0.9}}. This way, the resulting train set has a much heavier imbalance than the one used for the teacher. Figure 4 gives an overview of the distribution of classes for the teacher train set and the student and baseline train set.

Similar to the previous subsection, we train the teacher and the upper bound network by sampling 5000 images from the imbalanced 2cMNIST train set, using the fractions in Figure 4 (top). Next, we use the fractions in Figure 4 (bottom) to sample colored subsets of different sizes (10, 50, 100, 250, 500, 750, or 1000) to train the student and baseline networks. We evaluate all networks using the (balanced) 2cMNIST test set.

**Figure 5:**
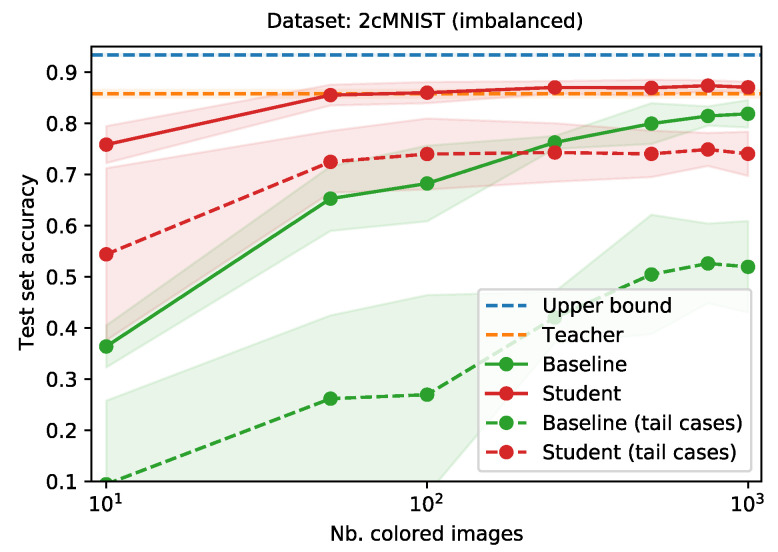
Comparison of the test set accuracy when training a neural network, with and without knowledge distillation, as a function of the train set size, for a heavily imbalanced train set. The dotted lines represent test set accuracy for the tail cases only, i.e., the digits “7”, “8” and “9”. Averages over three runs. Shaded areas indicate the standard deviation.

Figure 5 gives the performance of the teacher network (orange dotted line) and upper bound (blue dotted line), as well as the performance of the student network, trained using distillation (red) and baseline network (green) as a function of their train set size. Without the additional information provided by the teacher, the baseline struggles to achieve similar performance. Even when the train set grows, the baseline network never catches up to the student network (trained using knowledge distillation). The student quickly matches the performance of the teacher. The difference in performance is the most explicit in the tail classes. The dotted green and red lines show the test set accuracy when only looking at the digits “7”, “8” and “9” (the least represented digits in the train set). For these classes, the baseline network never manages to learn anything meaningful. The student network, on the other hand, manages to achieve reasonable performance when trained with 50 or more images.

### 4.3. Extensions

Next, we evaluate our proposed extensions. These are (i) annealing α, and (ii) selective knowledge distillation. For this, we use the dual-modality CIFAR-10 dataset. Here, the greyscale images represent the old data modality, and the colored images represent the new modality (similar to the 2cMNIST dataset).

#### 4.3.1. Annealing α

We evaluate our first proposition, annealing α, by training a series of teacher and student networks, where the student is trained using knowledge distillation, varying both the starting and ending values for α. This way, we create scenarios where either the distillation loss or the student loss predominates training, or where the influence shifts from teacher to student as training progresses. For comparison, we train upper bound and baseline networks as well, similar to the previous subsection.

We use the same architecture for all networks, consisting of three convolutional layers. Each of these layers is followed by a ReLU non-linearity, and a max pooling operation with a kernel of size 2×2. The convolutional layers all have a kernel size of 3×3, and have 64, 128 and 256 filters, respectively. The convolutional layers are followed by a dropout operation, with a probability of 0.5. The networks end in the classification layer, which is a fully connected layer, mapping the hidden representation to the ten output classes.

For all networks, training is carried out for 50,000 iterations, using a batch size of 64. Network parameters are updated using stochastic gradient descent, with a learning rate of 0.01, and a momentum of 0.5. The full train set of 50,000 images is used to train the teacher (using the greyscale versions of the images) and the upper bound (using the colored versions). We sample a random subset of 5000 images to train the student and baseline networks.

**Figure 6:**
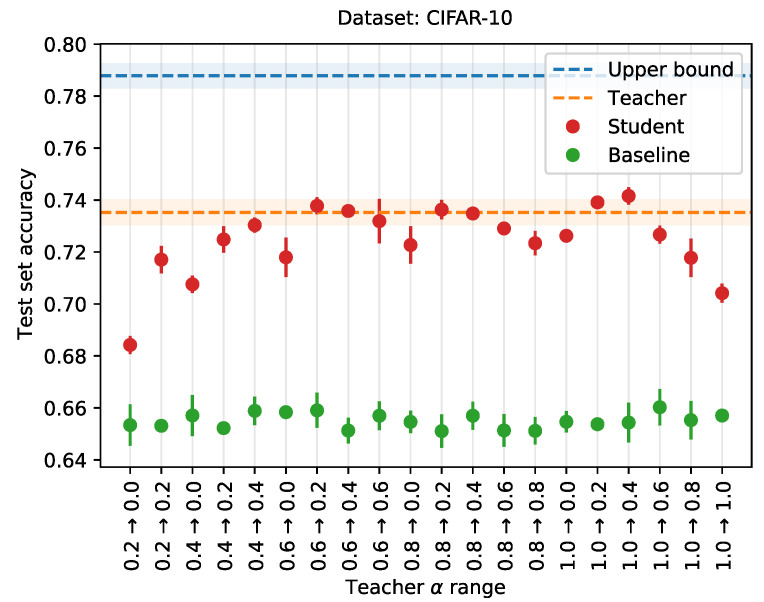
Training a student network with knowledge distillation, with linearly annealing values for hyperparameter α. The range for α is given on the x-axis, while the y-axis gives the test set performance. Averages over three runs. Shaded areas and error bars indicate the standard deviation.

Figure 6 gives the performance of the student network (red), trained using knowledge distillation with α-annealing, compared to the baseline network (green), trained without knowledge distillation. The performance of the teacher is given by the orange dotted line. The range over which α is annealed is marked on the x-axis. Annealing α improves performance, although not substantially. Neither the extremes, relying too little on the teacher (e.g., range 0.2→0.0), or relying too much on the teacher (e.g., range 1.0→0.8), are beneficial to the student’s performance. Additionally, discarding the teacher’s knowledge towards the end of training, i.e., ranges going to zero, appears detrimental as well. Hence, the initial value for α must be high enough, i.e., in [0.6, 1.0], as to bootstrap the student with the teacher’s knowledge, and the final value must be low enough, although not too low to allow the student to discard the teacher’s knowledge, i.e., in [0.2, 0.4].

**Figure 7:**
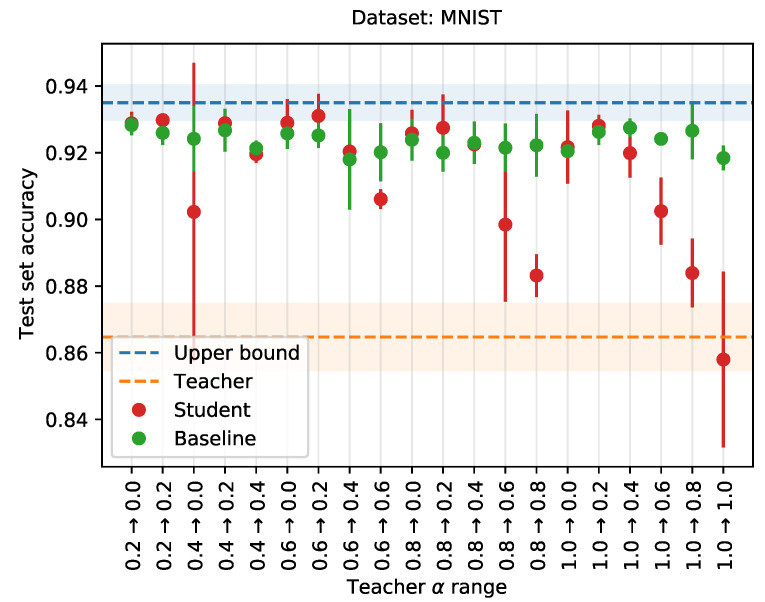
Training a student network with knowledge distillation, with linearly annealing values for hyperparameter α, on the 2cMNIST dataset. The range for α is given on the x-axis, while the y-axis gives the test set performance. Averages over three runs. Shaded areas and error bars indicate the standard deviation.

Following these results, we revisit the 2cMNIST experiment in Section 4.2. Without annealing, performance of the student seems to stagnate after a given threshold. Moreover, the baseline surpasses the student given a large enough train set. This could indicate that the influence of the teacher undermines the performance of the student. We verify this hypothesis by evaluating the different annealing schemes for the final data point, for a train set size of $10^{3}$. Figure 7 shows the results. We see that, indeed, following the recommendations above for annealing α, the student manages to outperform the baseline.

#### 4.3.2. Selective Knowledge Distillation

To evaluate this method, we train a teacher network for the full 50,000 train steps. The teacher achieves an accuracy of 0.77 on the student (train) subset. Next we train a student network using (default) knowledge distillation for 25,000 steps (half the training, i.e., the bootstrapping phase). We duplicate this student, and continue training the original version for another 25,000 steps. The duplicate version is trained using selective knowledge distillation.

**Figure 8:**
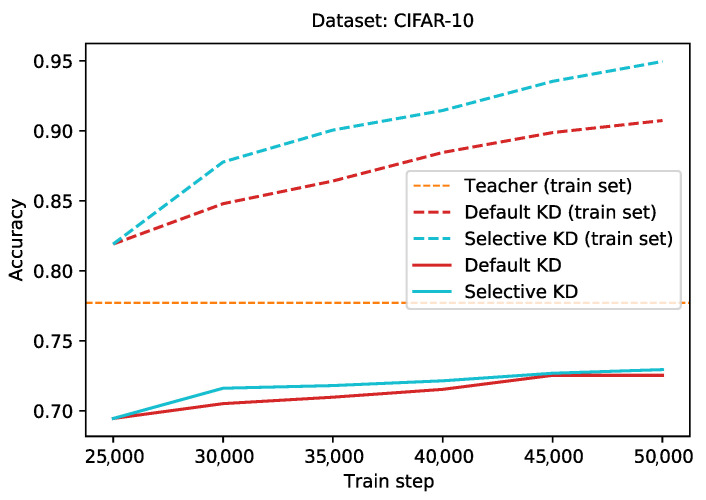
Intermediate performances of a student network trained using default knowledge distillation, and a student trained using selective knowledge distillation, starting at the middle of training. Full lines represent test set accuracy, while the dotted lines stand for train set accuracy.

Figure 8 gives an overview of train (dotted line) and test (full line) set performance for the second half of training, both for the student trained with default knowledge distillation (red), and the student trained with selective knowledge distillation (cyan). At the end of training, the selective student achieves a higher train set performance, reaching a train set accuracy of 0.95, compared to an accuracy score of 0.91 for default knowledge distillation. However, this only slightly transfers to the test set, where the difference in accuracy between both approaches is less than 0.01. By discarding teacher information in some cases, the student could overfit the train set faster.

### 4.4. Skin Lesion Classification

Finally, we apply our method to the real-world use case of (multispectral) skin lesion classification. In this last experiment, we compare the performance of a teacher network, that was trained on the larger ISIC dataset, to a baseline network and a student network, both trained on the smaller multispectral dataset. We use the EfficientNet-B0 [5] architecture for all networks, only varying the number of input planes. We train the teacher network using the ISIC train set. Training is done for 128 epochs, using a batch size of 32. A model checkpoint is stored after every epoch. We select the checkpoint that achieves the lowest loss on the ISIC validation set. This checkpoint scores an accuracy of 0.74 on the Demetra test set (white light). Following, we train a student and baseline network on increasing fractions of the Demetra train set (multispectral). We use the fractions {0.5,⋯,0.9,1.0}. For the student network, we distill knowledge from the teacher, with a value of 0.6 for α, and a temperature T=8. For each of the aforementioned fractions, we train for 128 epochs, serving batches of size 32. We use the validation set to choose the epoch checkpoint with the lowest validation loss. For the student network, we pass the white light image of a lesion through the teacher, to obtain soft labels. We train all networks with a learning rate of 0.001. Parameters are updated using stochastic gradient descent, with a momentum of 0.9.

**Figure 9:**
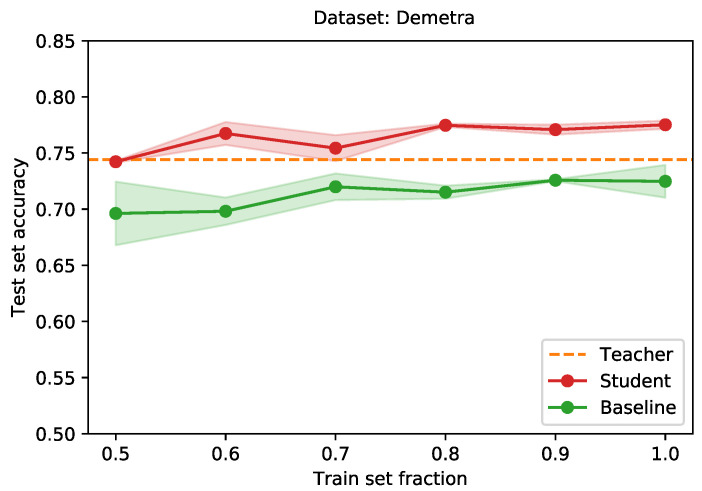
Comparison of the average test set accuracy when training a neural network, with and without knowledge distillation, as a function of the student train set size. The teacher network is trained using the ISIC train set, of 24,064 images. The student and baseline networks are trained using fractions of the Demetra train set (having a total of 5156 images). Averages over three runs. Shaded areas indicate the standard deviation.

Figure 9 shows the test set accuracy for the student (red) and baseline (green) networks, for each of the fractions. The performance of the teacher is given by the orange dotted line. Even for the smaller fractions, the student manages to achieve a competitive performance. As more data becomes available, the student surpasses the teacher.

## 5. Discussion

When upgrading sensors to a new, more informative, data modality, a large dataset in the new modality is usually unavailable. This could potentially hurt performance in a classification setting, where a neural network was previously trained on a large body of data in the old modality. In this work, we have evaluated cross-modal distillation to alleviate this hard transition, by employing the previously trained network as a teacher network, to supervise training of a student network on the limited dataset in the new modality.

We have validated this approach using a modified version of the MNIST dataset of handwritten digits, named 2cMNIST. By coloring hard to distinguish digits in two different colors, we have introduced an additional degree of freedom in the data. This simulated the upgrade path to a more informative data modality. We trained a teacher network on the greyscale version of the images, and a baseline and student network on the colored version, varying the amount of color images available. For the student network, we distilled knowledge from the teacher network. Even when only 10 images were available for the student, this network manages to achieved a test set accuracy of 0.77, compared to an accuracy of 0.37 for the baseline. This gap reduced as more data became available. For 100 images, the student and baseline network achieved a test set accuracy of, respectively, 0.90 and 0.86.

The benefits of cross-modal distillation became even more clear when introducing a class imbalance in the 2cMNIST dataset, especially for the tail cases, i.e., the digits that were least represented in the train set. We repeated the previous experiment using an imbalanced version of the 2cMNIST dataset. For the tail cases, the student managed to achieve a competitive test set accuracy of 0.74 when trained from 100 images or more. On the other hand, even with 1000 training images, the baseline reaches a test set accuracy no higher than 0.52.

We have proposed and evaluated two extensions to the default knowledge distillation scheme, using a modified version of the CIFAR-10 dataset. First, we presented a linear annealing scheme for the hyperparameter α. By decreasing the value for α over training, we reduced the influence of the teacher towards the end of training. Any annealing scheme resulted in the student network outperforming the baseline. Starting with a high enough value, i.e., in [0.6, 1.0], and ending with a low enough value, i.e., in [0.2, 0.4], achieved the best performance, with a test set accuracy of 0.74 for the range 1.0→0.4. This in comparison to, for example, an accuracy score of 0.68 for the range 0.2→0.0. Next, we proposed selective distillation, an approach where knowledge is only distilled from a teacher if the teacher was correct. While selective distillation also slightly increases performance, some caution is in order, for the student not to overfit on the train set.

Finally, we have applied our method to the real-world use case of multispectral skin lesion classification. For this, we used a large of white light images to train a teacher, and a smaller dataset of multispectral images to train a student and baseline network. Using knowledge distillation, the student manages to catch up to the teacher (even surpassing it), at a much faster rate than the baseline.

## 6. Concluding Remarks

In this work, we have proposed and evaluated cross-modal distillation, as well as two extensions, to reduce the impact of upgrading a sensor in a classification setting. We have validated this technique and the extensions using modified versions of the MNIST and CIFAR-10 benchmark datasets. Next, we applied our approach to the real-world problem of skin lesion classification. By leveraging the knowledge of a powerful teacher network, we could considerably improve performance of a student network, allowing for a much faster application of the upgraded sensor.

While the MNIST and CIFAR-10 dataset are well-established datasets in the deep learning community, both remain visual datasets, possibly limiting the validity of our approach. In future work, we plan to further evaluate our approach using different modalities, such as lidar and radar data. In addition, in our experiments, we chose neural network architectures achieving competitive performance, without performing a full search for the optimal hyperparameters. The effect of hyperparameters on the performance of cross-modal distillation therefore remains an open question we hope to target in future work.

## Figures and Tables

**Table 1:** Distribution of the labels in the Demetra dataset.

Label	Number of Images	Fraction of the Data
Melanoma	284	0.04
Nevus	4083	0.59
Basal cell carcinoma	891	0.13
Actinic keratosis/squamous cell carcinoma	641	0.09
Benign keratosis	653	0.09
Dermatofibroma	141	0.02
Vascular lesion	182	0.03

## Data Availability

Data used in these experiments can be found as follows:
MNIST: http://yann.lecun.com/exdb/mnist/ (accessed on 25 September 2021). CIFAR-10: https://www.cs.toronto.edu/~kriz/cifar.html (accessed on 25 September 2021). ISIC: http://challenge2019.isic-archive.com/ (accessed on 25 September 2021).
The Demetra dataset of multispectral skin lesions is proprietary and owned by Barco Healthcare. We therefore cannot share it
